# Additional Case of Croup, Successfully Treated with Digitalis

**Published:** 1800-11

**Authors:** G. Custance

**Affiliations:** Kidderminster


### *Additional Case of Croup, successfully treated with Digitalis.*

To the Editors of the Medical and Physical Journal.

Gentlemen,

I Had the honour in June laſt, to tranſmit to you two caſes of *Croup*, which I had ſucceſsfully treated with Digitalis, and which you had the goodneſs to inſert in No. XVII. of your uſeful Journal. The end I propoſed to myſelf in ſending them to you, was to call the attention of the profeſſion to the powers which the Digitalis apparently poſſeſſes of arreſting the dangerous ſymptoms of that dreadful diſorder. It doubtleſs has occurred to many gentlemen, as it did to me, to make trial of it, upon the principle of its operating ſo quickly and powerfully upon the arterial ſyſtem, and thereby ſtopping the rapid progreſs of the inflammatory ſymptoms. Conſidering the common fatality of the Croup, and the little command we have over it by the remedies hitherto uſually employed, I cannot but wiſh that the following additional caſe of Croup I now ſend you, may be a means of promoting a trial of the Digitalis. So much has been written by ingenious men on the ſubject of Croup, that it is quite needleſs for me to enter particularly into it. The definition of it given by Dr. Cullen in his

Synopſis,

Synopsis, is so truly characteristic, that it is scarcely possible for any person to mistake the disorder: the "vox rauca" and "tussis clangosa," will infallibly determine the real existence of Croup.

September 17, 1800, Elizabeth Clark, two years of age, was *suddenly* seized last night at eight o'clock with hoarseness and difficulty of breathing; both which symptoms are greatly increased this morning. She coughs with a barking noise; pulse very quick; belly natural.

Sumat tincturæ Digitalis, (secundum Dr. Maclean) gt. vj. 4tâ. quâque horâ.

18th. Has taken five doses of the drops; pulse not too frequent; dyspnœa and cough *entirely* gone. Sumat guttas omni 6tâ horâ.

19th. Took the drops regularly every six hours; had a slight return of dyspnœa and cough last night, which continued about an hour; slept well afterwards, and is this morning apparently well.

21st. Continues free from complaint.

If you think the above worthy a place in your Journal, I shall send you a faithful account of the result of any future trials I may have an opportunity of making of the Digitalis, as a remedy for the Croup; and beg leave to observe, that the three already sent, are the only cases of Croup which have fallen under my care, since it occurred to me to try the Digitalis.

The Vaccine Inoculation has been brought before the tribunal of the public with such a *weight* of evidence, that I think an *impartial* jury must give a verdict in its favour. It seems, therefore, quite superfluous to bring forward any more witnesses. Yet, as late experience has removed a doubt from my mind, which, probably, still lies as an objection against Cow-pox with many others, I think it my duty to state the following circumstance in favour of it, as I had *previously* an intention of communicating it to the public, had a *contrary* result taken place. About two months ago, I delivered the wife of Thomas W. a watch-maker in this town, of a girl, which I inoculated for the Small-pox about three weeks ago. Mrs. W. and two other young children, had been inoculated at Cheltenham, for the Cow-pox, by Dr. Jenner himself, about fourteen months ago, and afterwards for the *Small-pox*, which they told me did not take place in either of the three. Doubting whether time might not so far alter the constitution, at least of children, as to destroy the former good effects of Vaccine Inoculation, I inoculated them again with the infant. I am happy to ſay, it totally failed in all *three*, although the infant, who was inoculated with the ſame matter, ſickened, and had an unuſual number of puſtules. I am,

Your's, &c.

*Kidderminſter,*
*October* 12, 1800.

G. CUSTANCE.

